# Co-Processed Chitin-Mannitol as a New Excipient for Oro-Dispersible Tablets

**DOI:** 10.3390/md13041739

**Published:** 2015-03-30

**Authors:** Nidal Daraghmeh, Babur Z. Chowdhry, Stephen A. Leharne, Mahmoud M. H. Al Omari, Adnan A. Badwan

**Affiliations:** 1 The Jordanian Pharmaceutical Manufacturing Co., PO Box 94, Naor 11710, Jordan; E-Mails: nidalda@hotmail.com (N.D.); momari@jpm.com.jo (M.M.H.O.); 2 Faculty of Engineering & Science, University of Greenwich, Medway Campus, Chatham Maritime Kent ME44TB, UK; E-Mails: b.z.chowdhry@greenwich.ac.uk (B.Z.C.); s.a.leharne@greenwich.ac.uk (S.A.L.)

**Keywords:** crystalline mannitol, α-chitin, roll compaction, oro-dispersible tablets

## Abstract

This study describes the preparation, characterization and performance of a novel excipient for use in oro-dispersible tablets (ODT). The excipient (**Cop**–**CM**) consists of chitin and mannitol. The excipient with optimal physicochemical properties was obtained at a chitin: mannitol ratio of 2:8 (w/w) and produced by roll compaction (RC). Differential scanning calorimetry (DSC), Fourier transform-Infrared (FT-IR), X-ray powder diffraction (XRPD) and scanning electron microscope (SEM) techniques were used to characterize **Cop**–**CM**, in addition to characterization of its powder and ODT dosage form. The effect of particle size distribution of **Cop**–**CM** was investigated and found to have no significant influence on the overall tablet physical properties. The compressibility parameter (a) for **Cop**–**CM** was calculated from a Kawakita plot and found to be higher (0.661) than that of mannitol (0.576) due to the presence of the highly compressible chitin (0.818). Montelukast sodium and domperidone ODTs produced, using **Cop**–**CM**, displayed excellent physicochemical properties. The exceptional binding, fast wetting and superdisintegration properties of **Cop**–**CM**, in comparison with commercially available co-processed ODT excipients, results in a unique multifunctional base which can successfully be used in the formulation of oro-dispersible and fast immediate release tablets.

## 1. Introduction

Tablets are widely used as drug delivery system due to their convenience with respect to self-administration and ease of manufacture [1]. Various excipients are used in their preparation [2]. However, pediatric, geriatric and mentally ill patients experience difficulties in swallowing conventional tablets, which leads to poor patient compliance. To overcome this deficiency, ODT formulations have been developed [3,4].

In Ph. Euro., an ODT is defined as a tablet to be placed in the mouth where it disperses rapidly before being swallowed in less than 3 minutes [5], while the FDA considers it as a solid oral preparation that disintegrates rapidly in the oral cavity with an *in vivo* disintegration time of approximately 30 s or less [4]. ODTs are advantageous due to their administration without water, rapid onset of action and improved bioavailability [6,7].

Various ODT patented technologies such as Orasolv/DuraSolv (by direct compression (DC)), Zydis (by freeze drying), FlashTab (Eudragit-microencapsulation and effervescent couple), FlashDose (cotton candy process) and WowTab (compression moulding process) have been commercialized [8,9]. ODTs are highly friable, due to their compaction at lower crushing force compared to conventional tablets, resulting in rapid disintegration; therefore, they are commonly packed in special packaging materials, which add to their cost [10]. A high level of superdisintegrant (up to 20% w/w) is usually needed in ODT preparations in order to enhance their disintegration properties. Additional excipients including a suitable filler, binder, lubricant, sweetener and color may be added to improve product properties [6,11].

Mannitol, in crystalline, granulated, or spray dried form is widely used in ODT tablet formulations due to its sweet, cool taste and compatibility with a wide range of drugs [12,13,14]. Unlike crystalline mannitol, spray dried mannitol is highly compactible, non-friable, and quick dissolving, which facilitate its use in DC formulations of ODTs. Furthermore, crystalline mannitol is widely used in tablet wet granulation (WG) processes due to its favourable cost [15,16,17].

Chitin is used as chromatographic supports and adsorbents for industrial pollutants [18,19], for recovery of silver thiosulfate complexes [20], enzyme immobilization [21], wound healing [22], fibers and film formers [23], and binders in the paper making process [15]. Recently, chitin has been used as a starting material to produce pyranoside [24] and furan [25] derivatives. Furthermore, chitin monomer *N*-acetyl-d-glucosamine has been used as a source of amide/amino substituted sugar alcohols [26].

Chitin is used in tablet formulations because it is non-toxic, non-allergenic, anti-microbial, non-reactive and biodegradable. Its disintegration power is mainly dependent upon a high water uptake rate. Therefore, chitin can be used over a higher concentration range than many commercially available disintegrants without negatively affecting other tablet properties. However, chitin powder shows poor compactibility, which has limited its applications in commercialized dosage forms [27,28]. To overcome this shortcoming, chitin was co-processed with other excipient modifiers in order to facilitate its handling in solid dosage form manufacturing. As with other excipients, co-processing is carried out using different manufacturing techniques such as spray drying or melt extrusion [29]. For example, chitin, a plastic material, has been successfully used to prepare different excipients via co-processing with diverse brittle materials including mannitol, metal silicates and silicon dioxide [30,31,32]. In all cases co-processing was achieved by incorporating the brittle material (≤30% w/w) inside the pores of chitin (≥70% w/w) using an aqueous vehicle. The result of the foregoing studies showed that the extremely large surface pores of chitin measured by BET analysis [31,33,34] were not fully accommodated by the guest materials and thus chitin preserved its functionality as a disintegrant. Moreover, the co-processed excipients enhanced the physical properties, functionality and performance (e.g., no-hygroscopicity and highly compactable/disintegrable) of tablet preparations [30,31,32]. A combination of chitin, chitosan-alginate (1:1), and glycine was reported in preparation of a novel superdisintegrant [35]. The forgoing excipient was applied to formulate sulbutamol sulphate ODT by using direct compression. This may offer a valuable practical industrial addition in terms of superdisintegration and mechanical properties of ODT formulations. Furthermore, an article describing the characterization and application of such a novel co-processed excipient in immediate release tablet formulations has been reported in the literature [32]. In the aforementioned study, the tablets prepared by using the co-processed excipient chitin:mannitol (80:20 w/w) and different drugs displayed excellent chemical stability, binding, and disintegration properties. This may extend its application to ODTs [32].

The objective of the work reported herein is to test the possibility of using co-processed chitin-mannitol as a single excipient in producing ODTs. This may help to overcome the problem of low crushing strength inherited from other techniques and to use chitin for the first time as the main excipient allowing the use of conventional industrial machinery for the production and packaging of ODTs.

## 2. Results and Discussion

### 2.1. Selection of Processing Methods and Ratios for Co-Processed Chitin–Mannitol Excipient

**Figure 1:**
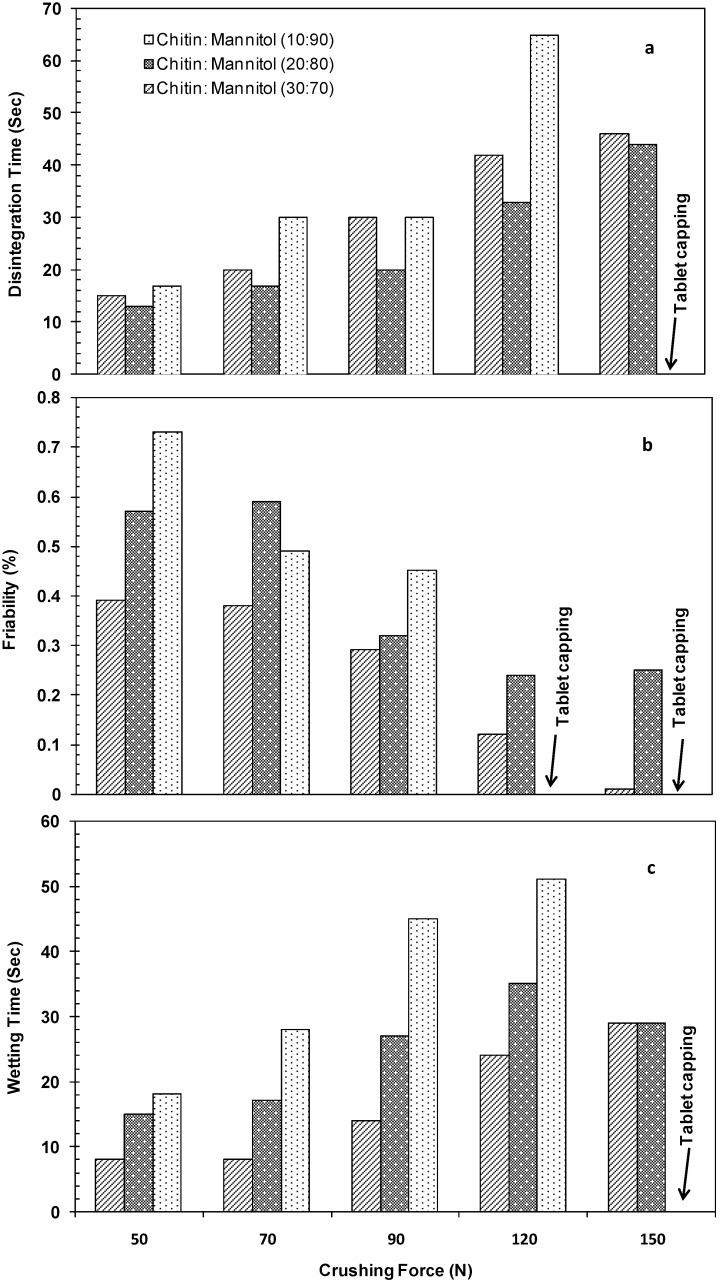
Plots of the crushing force (N) *versus* (**a**) disintegration time; (**b**) friability; and (**c**) wetting time for compacted mixtures prepared using different ratios of chitin and mannitol (1:9, 2:8, and 3:7 w/w). Tablets were 10 mm in diameter and 250 mg in weight. All powders were lubricated using 1% (w/w) sodium stearyl fumarate.

In order to select the optimal ratio and process for co-processed excipient preparation, three different ratios of chitin and mannitol (10:90, 20:80 and 30:70 w/w) and three different processing techniques *i.e.*, direct mixing, WG and RC were used. The prepared excipients were lubricated with sodium stearyl fumarate (1.0% w/w) and compressed at different tablet crushing forces (50–150 N). The tablets obtained were tested for friability, disintegration and wetting times *versus* the corresponding crushing forces. The preliminary results of the aforementioned experiments indicated that direct mixing and WG were unsuitable, because the mixtures prepared by direct mixing displayed unacceptable physical properties (e.g., poor flow and powder non-uniformity). The difference in bulk densities of chitin (~0.2 g/cm^3^) and mannitol (~0.5 g/cm^3^) is the reason underlying such unacceptable physical properties. In the case of WG, the tablets suffered from capping and high disintegration times due to penetration of the dissolved mannitol into the chitin pores. Such penetration does not allow the pores to act as functional compression and disintegration enhancers. However, RC gave reasonable results. The data in Figure 1 shows the effect of crushing force on the friability, disintegration and wetting times of tablets produced. Up to a crushing force of 90 N, all chitin:mannitol ratios showed acceptable physical properties (low friability and fast disintegration and wetting times). While at crushing forces above 90 N, the excipient prepared by using a chitin:mannitol ratio of 1:9 showed capping upon tablet compression due to an insufficient amount of chitin, responsible for improving the compressibility. Using chitin:mannitol ratios of 2:8 and 3:7 (w/w) over all the investigated range of crushing forces produced tablets with acceptable physical properties. However, a ratio of chitin and mannitol of 2:8 (w/w) was chosen in order to obtain beneficial mannitol taste properties and to reduce the amount of insoluble chitin in ODT preparations.

### 2.2. Characterization of **Cop–CM** Powder

**Figure 2:**
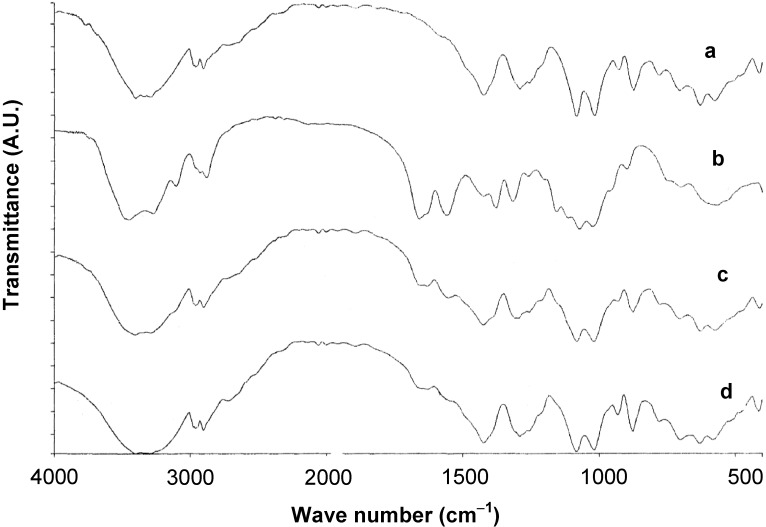
FT-IR spectra of (**a**) mannitol; (**b**) chitin; (**c**) physical mixture of chitin-mannitol (2:8, w/w); and (**d**) co-processed chitin-mannitol (2:8 w/w) excipient (**Cop–CM**).

The FT-IR spectra of chitin, mannitol, the corresponding physical mixtures and **Cop-CM** are presented in Figure 2.

It is clear that the FT-IR spectrum of the physical mixture of chitin and mannitol (Figure 2c) is a superimposition of the vibrational band profiles contributed by chitin and mannitol (Figure 2a,b). The dominance of the principal bands of mannitol in the physical mixture is a result of its high fractional composition in the mixture (80% w/w). The two bands in the 1550–1660 cm^−1^ range, corresponding to the amide I and II vibrational modes of chitin (Figure 2a), persist in the spectra of the physical mixture and **Cop–CM**, while the remaining bands are due to mannitol. The absence of any shift in the FT-IR bands of **Cop–CM** (Figure 2d), in comparison with the bands of the physical mixture (Figure 2c), suggests the absence of chemical interaction due to the use of RC to form **Cop–CM**.

**Figure 3:**
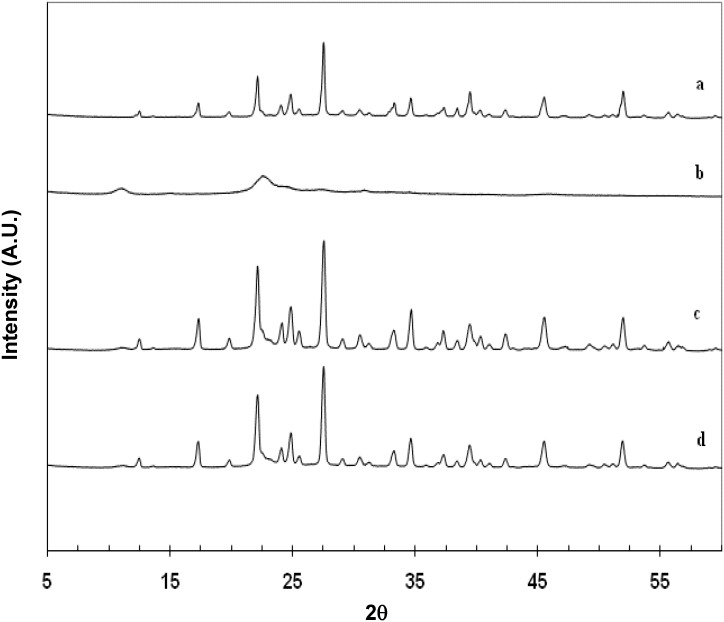
XRPD profiles of (**a**) mannitol; (**b**) chitin; (**c**) physical mixture of chitin-mannitol (2:8, w/w); and (**d**) co-processed chitin-mannitol (2:8 w/w) excipient (**Cop–CM**).

**Figure 4:**
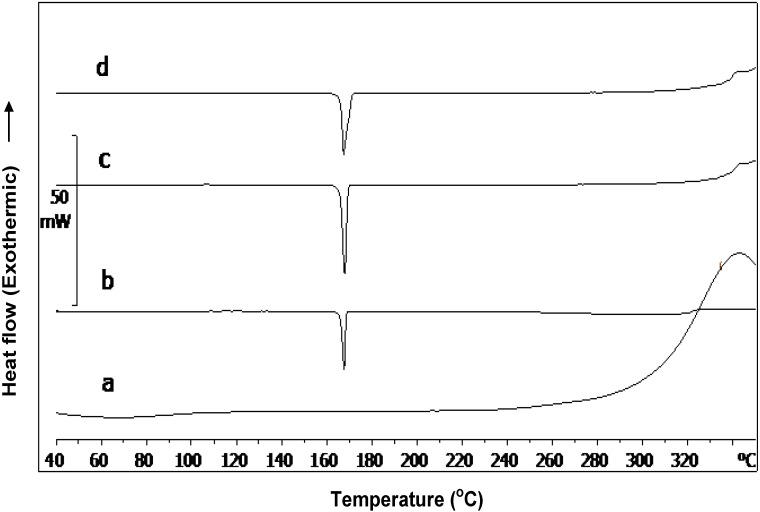
DSC thermograms of (**a**) chitin; (**b**) mannitol; (**c**) physical mixture of chitin-mannitol (2:8, w/w); and (**d**) co-processed chitin-mannitol (2:8 w/w) excipient (**Cop–CM**).

Further analysis of **Cop–CM** by XRPD (Figure 3) and DSC (Figure 4) techniques showed the same results, where the signals corresponding to mannitol are dominant due to its high fractional content and crystallinity. The two broad peaks of chitin appeared at 2θ of around 11 and 22 but not clearly well-defined in both the physical mixture and **Cop–CM** due to the low content of chitin in the mixtures. The absence of new bands or shifts in the patterns indicates the absence of formation of a new crystal form or chemical interaction. This property is essential for such co–processed excipient to work as an excipient.

**Figure 5:**
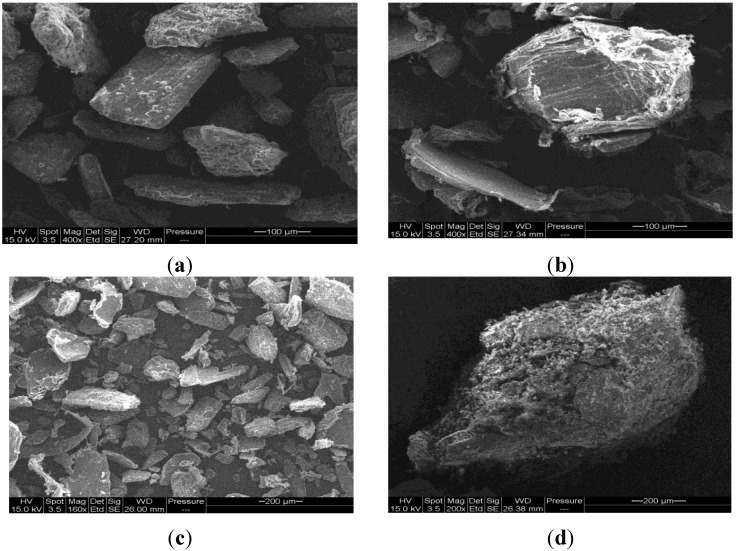
SEM images of (**a**) mannitol; (**b**) chitin; (**c**) physical mixture of chitin-mannitol (2:8, w/w); and (**d**) co-processed chitin-mannitol (2:8 w/w) excipient (**Cop–CM**).

SEM was used to investigate particle surface morphology (Figure 5). When comparing the particle shape of mannitol (Figure 5a) with that of **Cop–CM** (Figure 5d), it is apparent that it has changed from rectangular rod granules to three-dimensional dense compacts for **Cop–CM**.

### 2.3. Physical Properties of **Cop–CM** Powder

The physical properties of **Cop–CM** powders which passed through two different mesh size sieves (710 and 1000 μm) were evaluated. The two powders have a water content of about 1.5% w/w and a bulk density of 0.5–0.55 g/cm^3^. The pH of a 5% (w/v) aqueous dispersion is in the range 6–8. The particle size distribution (D10, D50 and D90) of **Cop–CM** is given in Table 1.

Particle size and shape are critical parameters in powder characterization, particularly in DC formulations affecting powder performance, packing, consolidation, flowability and compaction. It is one of the prime considerations in selecting excipients to develop and optimize a pharmaceutical formulation. Ideally, DC excipients should exhibit narrow size distributions with moderate-to-coarse particle, having a mean particle size of 100–200 μm [36]. In ODT formulations, the control of particle size is essential for the water insoluble excipients to minimize the feeling of grittiness during tablet administration. The best results are obtained when using a smaller particle size for insoluble excipients. Another critical parameter for the DC excipient is the bulk density which can be used to describe the packing behavior of granules [36]. **Cop–CM** powder passed through a mesh size of 710 μm has a mean particle size of about 215 μm. A higher bulk density is advantageous in tabletting because of a reduction in the powder-fill volume of the die. **Cop–CM** powder sieved through a mesh size of 1000 μm has a bulk density of about 0.52 gm/mL and a tapped density of 0.59 gm/mL.

**Table 1:** The physical properties of co-processed chitin-mannitol (2:8 w/w) excipient (**Cop–CM**).

Parameter	Value
Water content (w/w%)	1.5
pH	6.0–8.0
Bulk density (gm/mL)	0.50–0.55
Tapped density (gm/mL)	0.55–0.65
Particle size distribution:	
- Milling through 710 μm	D10: 5 μm; D50: 145 μm; D90: 496 μm
- Milling through 1000 μm	D10: 7 μm; D50: 170 μm; D90: 584 μm
Hausner Ratio	1.13
Carr Index	11.86
Angle of Repose	32°

On the other hand, Hausner Ratio (*H*) is an indirect index of ease of powder flow. It is calculated by using the formula [37]

(1) $$H=\frac{TD}{BD}$$

where TD and BD are the tapped and bulk densities, respectively.

The simplest method of measurement of free flow of a powder is compressibility; an indication of the ease with which material can be induced to flow is given by Carr Index (*I*) which is calculated using the formula:

(2) $$I=100\frac{\left(TD-BD\right)}{TD}$$

Carr Index of ≤10 indicates excellent flow, whereas *I* values of 11–15 indicate good flow. Hausner Ratio of 1.0–1.11 indicates excellent flow whereas values of 1.12–1.18 indicate good flow (generally lower Carr Index and Hausner Ratio values represent better flow).

The change in density before and after tapping calculated as % compressibility (Carr Index) is an indicator of how fast granules can flow to their highest packing. The Carr Index calculated from the density data showed a value less than 12, and Hausner ratio of less than 1.13 further indicating the good flowability, which is an important factor for DC powders. Good flowability of powder is needed for content uniformity and less weight variation in the final tablets. According to US Pharmacopeia 31, General Chapter <1174>, an Angle of Repose of 25–30° indicates excellent flow, and 31–35° indicates good flow. The Hausner Ratio, Carr Index and Angle of Repose values for **Cop–CM** powder are shown in Table 1. From the data obtained, **Cop–CM** powder showed good flowability and compressibility.

### 2.4. Moisture Uptake by **Cop–CM**

**Figure 6:**
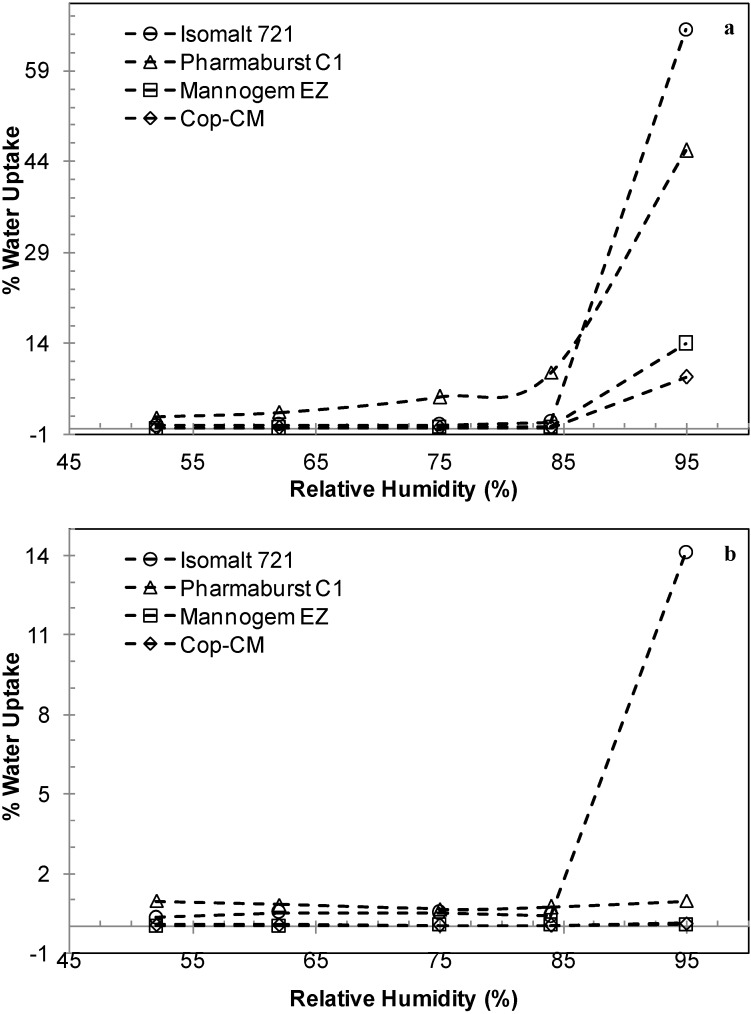
The water uptake by co-processed chitin–mannitol (2:8 w/w) excipient (**Cop–CM**) in comparison with some commercially available ODT bases after incubation at 25 °C and different relative humidity, (**a**) for two weeks; and (**b**) after equilibration at 25 °C/45% relative humidity for one further day.

The data in Figure 6 shows the water uptake by **Cop–CM** and commercial ODT bases (Phrmaburst C1, Isomalt 721, and Mannogem EZ) at 25 °C and different relative humidity. With up to 84% relative humidity for two weeks, all bases, except Pharmaburst C1, showed an insignificant increase in water uptake (Figure 6a). However, following equilibration for one day at 25 °C/45% relative humidity, Pharmaburst C1 lost the excess water absorbed (9%) to reach only 0.7% (Figure 6b). At high relative humidity (95%), the water uptake follows the order: **Cop–CM** (8%) < Mannogem EZ (14%) < Pharmaburst C1 (46%) < Isomalt galenIQ™ 721 (66%). Following equilibration for 1 day at 25 °C/45% relative humidity, all bases except Isomalt galenIQ™ 721 lost the excess water absorbed to give values less than 1% (Figure 6b). The relative humidity of Isomalt galenIQ™ 721 it is about 14% which is most probably due to partial dissolution of Isomalt at high relative humidity.

The difference in water uptake of different bases is due to the difference in their components and their morphology (e.g., crystalline *versus* amorphous). It seems that the RC of a large amount of mannitol (80% w/w) with chitin results in the coverage of the outer surface of chitin with mannitol; thus, **Cop–CM** powder is clearly non-hygroscopic due to the very minimal water uptake of mannitol.

### 2.5. Hygroscopicity of Tablets Prepared from **Cop–CM**

The water adsorption study was conducted for tablets prepared from **Cop–CM** at 75% relative humidity and room temperature. Results indicate that the tablets prepared from **Cop–CM** sorb very little, if any, water (less than 0.4% w/w). It is advantageous, in the pharmaceutical industry, to use non-hygroscopic ODT preparations, as this will reduce the cost of packaging which is usually used for moisture protection. Needless to say, wetting and ability to disintegrate must prevail.

### 2.6. Compression Profile of Tablet Prepared from **Cop–CM**

**Figure 7:**
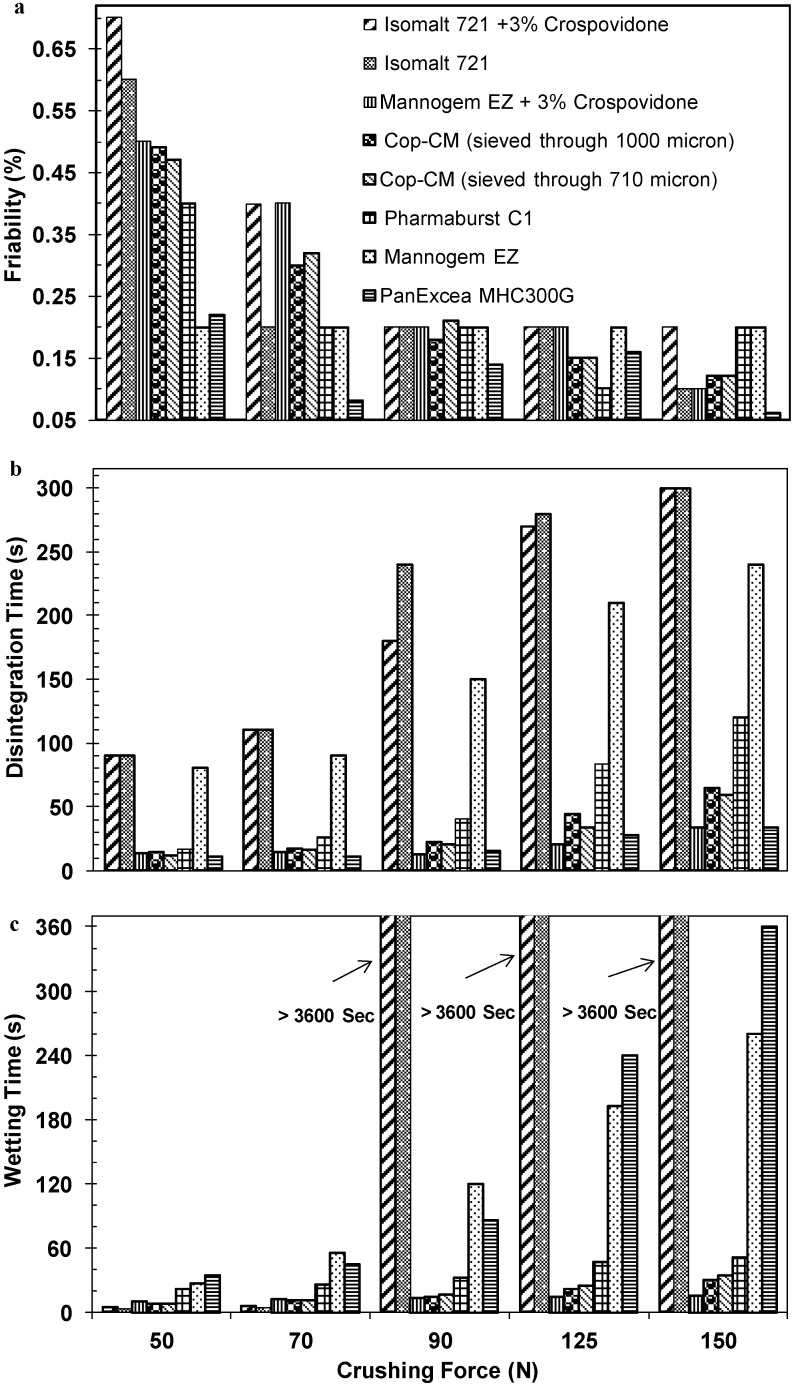
Plots of the crushing force (N) *versus* (**a**) friability; (**b**) disintegration time; and (**c**) wetting time for tablets prepared using co-processed chitin-mannitol (2:8 w/w) excipient (**Cop–CM**) which passed through either 710 μm or 1000 μm sieves in comparison with commercially available bases. The tablets were 10 mm in diameter and 250 mg in weight. All samples were lubricated with 1% (w/w) sodium stearyl fumarate.

Data for the friability, disintegration and wetting times *versus* the corresponding crushing force for tablets prepared using **Cop–CM** powders which passed through either the 710 μm or 1000 μm sieves are shown in Figure 7. Generally, a reduction in particle size is associated with an increase in tablet mechanical strength. The increase in mechanical strength of the tablets is directly related to its physical properties (crushing force, disintegration time, friability, wetting time, *etc.*) and attributed to an increase in the surface area available for inter-particulate attraction [38]. However, when **Cop–CM** was used it was found that varying the particle size had no impact on the mechanical strength of the tablets. The mechanical strength of the tablets prepared from materials with a tendency to fragment, such as mannitol, dibasic calcium phosphate dihydrate and saccharose appear to be independent of particle size [39]. This could be the case for **Cop–CM** particles which contain an excess amount of mannitol (80% w/w) and undergo fragmentation [40] at the early stages of compression, thereby causing a minimal effect on tablet mechanical strength when varying the particle size.

The disintegration time of ODTs is generally less than one minute and the actual disintegration time that patients expect is less than 30 s. The general compendial method for performing disintegration tests for ODTs is not capable of detecting such a short disintegration time. The wetting time of the ODT is another important test, which gives an insight into the disintegration properties of the tablets. A lower wetting time indicates a quicker disintegration of the tablets [41]. With respect to tablet disintegration, **Cop–CM** showed a unique characteristic whereby tablet disintegration time was independent of particle size and tablet crushing force (Figure 7). Tablets produced from **Cop–CM** powders which passed through either the 710 μm or 1000 μm sieves, at an upper punch compression scale of 16–20 kN for each particle size, showed a superior disintegration time ranging from 11.5 to 59 s and from 14 to 64 s, respectively. This was achieved for tablet crushing force values ranging from 50 to 150 N. By increasing the crushing strength from 50 to 150 N, the wetting time of the tablet was increased from 9 to 35 s. This suggests that capillary action is the dominant mechanism for the disintegration of **Cop–CM** which is irrespective of the tablet crushing force [32]. In addition, the inter-particulate voids within the chitin particles in **Cop–CM**, as previously mentioned, remain intact and unchanged after using the RC procedure with mannitol and by varying the powder particle size. With respect to powder compressibility, chitin within **Cop–CM** mixture provides an effective means of obtaining hard tablets with low friability while persisting in its fast disintegration and wetting properties, as can be concluded from the data shown in Figure 7.

Four commercially available ODT excipients were used for comparison purposes including, Isomalt galenIQ™ 721, Mannogem EZ, Pharmabust EZ and PanExcea MHC200G. The data in Table 2 shows the function and composition of these excipients. Crospovidone, a cross-linked polymer of *N*-vinyl-2-pyrrolidinone, is often used at concentrations up to 5.0% [11]. In this study, it was used as disintegrant in tablet preparations at 3% w/w level in both Isomalt galenIQ™ 721 and Mannogem EZ powders. Both PanExcea MC200G and Pharmaburst C1 already contain calcium silicate and crospovidone as disintegrant in their compositions, respectively.

**Table 2:** Function and composition of commercial oro-dispersible tablet excipients.

Trade Name	Composition	Manufacturer	Advantages & Function
PanExcea MC200G	Mannitol (75%), calcium silicate (25%). Particle size: 50% (103 μm)	Avantor Performance Materials, Inc./Center Valley, PA, USA http://www.avantormaterials.com/	High performance, rapid disintegration, direct compression excipient for oro-dissolving tablets formulation
Mannogem™ EZ	Spray dried direct compression mannitol Particle size: 60% (75–150 μm)	SPI Pharma™, Inc., New Castel, DE, USA http://www.spipharma.com	Assist in formulating difficult to use non-hygroscopic ODT containing fine drugs
Pharmaburst ™ C1	Mannitol 84%, crospovidone 16%, silicon dioxide <1%		High compactibility, high loading in small diameter tablets, smooth mouth feel, rapid disintegration
Isomalt galenIQ–721	1-*O*-d-glucopyranosyl-d-mannitol dehydrate and 6-*O*-d-glucopyranosyl-d-sorbitol (1:3) Particle size: 90% (360 μm), 50% (220 μm)	BENEO–Palatinit GmbH (Mannheim, Germany) http://www.beneo–palatinit.com/en/Pharma_Excipients/galenIQ/galenIQ_Grades/galenIQ721/	Highly soluble agglomerated spherical isomalt for fast dissolving and very fast disintegrating direct compression tablet preparations

In order to obtain a tablet crushing force in the range of 50–150 N, upper punch compression scales of 25.50–29.5 kN were applied to all excipients except for PanExcea in which case a scale range from 39 to 42 kN was applied. For all reference excipients used, increasing the crushing force from 50 to 150 N leads to a linear increase in the disintegration and wetting time of the tablets. The friability of the tablets was found to be within the acceptable limit [42] (less than 0.7% for Isomalt galenIQ™ 721 and 3% crospovidone) at the lower crushing force (50 N). Increasing tablet crushing force leads to decrease the friability (less than 0.2%). The reference excipients except for Mannogem EZ and Isomalt galenIQ™ 721 (with and without crospovidone) gave a disintegration time of less than 30 s at 50 N tablet crushing force. In addition, the wetting time for all reference excipients was less than 30 s at the same crushing force. The data in Figure 7 clearly shows that by increasing the tablet crushing force, the disintegration times and wetting times were increased accordingly. Only, Isomalt galenIQ™ 721 plus crospovidone and PanExcea MC200G tablets showed a short disintegration time (less than 33 s) at the highest crushing force (150 N), whereas for the other excipients a disintegration time range of 120–300 s were observed. At a tablet crushing force of 150 N, only pharmaburst C1 and Mannogem EZ plus crospovidone showed a short wetting time of less than 60 s (52 and 16 s, respectively).

For **Cop–CM**, it can be clearly observed that increasing the tablet crushing force up to 150 N does not affect both disintegration and wetting times. This property is extremely advantageous where hard tablets with very fast disintegration can be prepared using **Cop–CM** and simply packed in traditional packaging materials. This prevents the need of a special type of packaging to avoid the breakage of the tablets during removal from the package. A combination of Mannogem™ EZ and 3% crospovidone has showed similar behavior to **Cop–CM** at high tablets crushing forces. PanExcea MHC300G has preserved the very short disintegration time at 150 N tablet crushing force, while the tablet wetting time was increased. On the other hand, Pharmaburst C1 behaves in a different manner, whereby increasing the tablet crushing force to 150 N the disintegration time was relatively high (>2 min), while the tablet wetting time was not affected.

### 2.7. Powder Compressibility

The Kawakita equation (Equation (3)) is used to study powder compression using the degree of volume reduction, C. The basis for the Kawakita equation for powder compression is that particles subjected to a compressive load in a confined space are viewed as a system in equilibrium at all stages of compression, so that the product of the pressure term and the volume term is a constant [43]:

(3) $$C=\frac{\left(V_{0}-V\right)}{V_{0}}=\frac{abP}{1+bP}$$

where, *V_o_* is the initial volume and *V* is the volume of powder column under an applied pressure, *P*. The constants *a* and *b* represent the minimum porosity before compression and plasticity of the material, respectively. The reciprocal of *b* defines the pressure required to reduce the powder bed by 50% [44,45]. Equation (3) can be re-arranged in linear form as:

(4) $$\frac{P}{C}=\frac{P}{a}=\frac{1}{ab}$$

The expression for particle rearrangement can be affected simultaneously by the two Kawakita parameters *a* and *b*. The combination of these into a single value, *i.e.* the product of the Kawakita parameters (*ab*), may hence be used as an indicator of particle rearrangement during compression [46].

**Figure 8:**
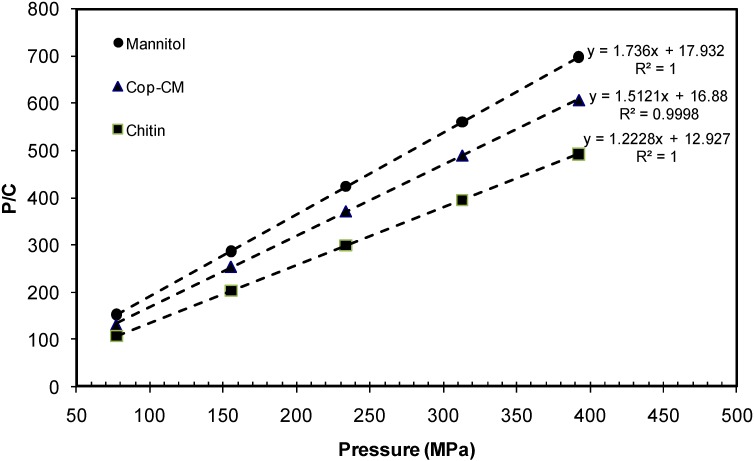
Kawakita plot for co-processed chitin-mannitol (2:8 w/w) excipient (**Cop–CM)**. Tablets were 12 mm in diameter and 400 mg in weight.

Figure 8 shows the Kawakita plots for mannitol, **Cop–CM** and chitin. The Kawakita constants *a, b, ab* and 1/*b* were calculated from the intercept and slope of the plots (Table 3). The constant *a*, which represents the compressibility, is the highest for chitin (*a* = 0.818) and this is due to the large internal surface pores. The compressibility of **Cop–CM** is higher than mannitol alone (*a* = 0.661 and 0.576, respectively) and this is ascribed to the addition of the chitin to mannitol. This result emphasizes the fact that although the mannitol constitutes 80% of **Cop–CM** content, using RC techniques in the preparation of **Cop–CM** keeps the large chitin surface pores unoccupied and active. This is because mannitol physically adheres at the outer chitin surfaces.

The increase in the *ab* value for **Cop–CM** (0.0592), which is a measure of the extent of particle rearrangement, indicates that the addition of chitin has improved the degree of particle rearrangement and packing during tabletting. The 1/*b* parameter is an inverse measure of the amount of plastic deformation occurring during the compression process [31,47]. Generally, a low value of 1/b is a reflection of the soft nature of the material and that the material is readily deformed plastically under pressure [48]. Chitin is a highly porous material and forms intermolecular hydrogen bonds between adjacent plastic, deformed chitin particles. The presence of moisture within the porous structure of chitin enforces the formation of hydrogen bond bridges which increase the internal binding upon compaction. Therefore, the use of a smaller amount of chitin (20%) with mannitol within **Cop–CM** decreases plastic deformation during compression [31,49].

**Table 3:** Kawakita parameters for mannitol, chitin and co-processed chitin-mannitol (2:8 w/w) excipient (**Cop–CM**).

Material	Kawakita Parameters					
	Slope	Intercept	*a*	*ab*	*b*	1 */b*
Mannitol	1.736	17.932	0.576	0.0558	0.0968	10.330
Chitin	1.223	12.927	0.818	0.0774	0.0946	10.570
**Cop–CM**	1.512	16.880	0.661	0.0592	0.0896	11.164

### 2.8. Loading Capacity

**Figure 9:**
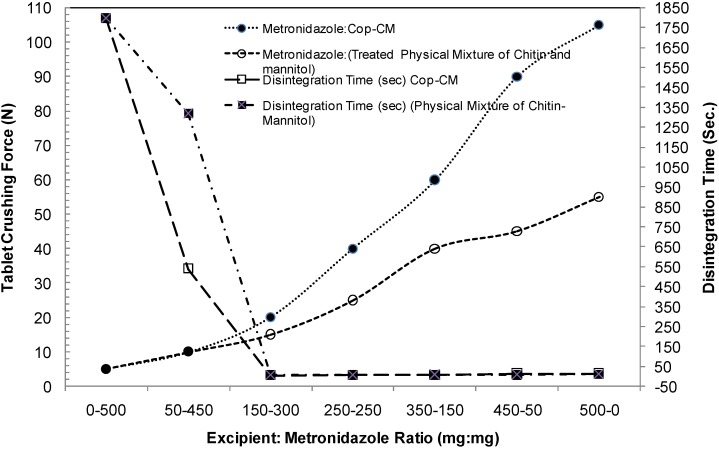
Relationship between tablet crushing force and disintegration time at different **Cop–CM**: metronidazole ratios using a physical mixture of chitin-mannitol (2:8, w/w) as a reference. Data are represented as the mean of *n* = 10. **Cop–CM** represents the co-processed chitin-mannitol (2:8 w/w) excipient.

The loading capacity of **Cop–CM** excipient was studied by using metronidazole as a model drug for an incompressible material. Compressing metronidazole alone (100% as a reference) gives rise to tablets with low mechanical strength and long disintegration times. However, the effect of increasing the weight ratios of **Cop–CM**/metronidazole from 0/500 to 500/0 (wt/wt) on these properties were investigated. The results indicated that by increasing the quantity of **Cop–CM** in the matrix, compactability is improved and the disintegration time is decreased as shown Figure 9. As a result, **Cop–CM** is capable of accommodate poorly compressible drugs with high loading capacity without affecting the physical and mechanical properties.

### 2.9. Functionality

The functionality of **Cop–CM** as an excipient was investigated by examining different processing techniques including DC, RC and WG to prepare tablets. The three formulations containing **Cop–CM** and low strength drugs (montelukast and domperidone) were investigated. Different reported ODT formulations for these two drugs using almost DC technique are summarized in Table 4 [50,51,52,53,54,55,56]. In these formulations, several excipients were used to obtain fast disintegrating tablets. Mannitol and microcrystalline cellulose were used as diluents, in addition to deferent superdisintegrants such as croscarmellose sodium, crospovidone, sodium starch glycolate and starch. Combination of talc and magnesium stearate was used as a lubricant system [51,52,54,56]. Furthermore, co-processing of two disintegrants, e.g., crospovidone and sodium starch glycolate, using alcoholic solvent was proposed to obtain fast disintegrating tablets [51]. DC followed by sublimation at 60 °C was also proposed for ODT formulation [56]. In the present work, co-processed mixture of mannitol and chitin by RC (**Cop–CM**) is used as a multi-functional excipients (diluents and superdisintegrant), in addition to limited number of excipients (flavor, sweetening agent) and sodium stearyl fumarate as inert lubricant. Although a limited number of excipients were used with **Cop–CM** as ODT base to improve the taste and to prevent sticking of powder on punches and dies during tablet compression, the fast disintegration properties (≤30 s) was attained at relatively high tablet crushing force (crushing force: 60–80 N) values (Table 5). The tablets obtained from all the processing techniques showed high internal binding and friability <0.4% (Table 5). The results indicate that **Cop–CM** is compressible and preserves its functionality whether utilized in DC, RC or WG formulations. Hence, it can be used as a multifunctional base (binder, filler and disintegrant) in ODT formulations.

**Table 4:** Composition of various ODT formulations of montelukast sodium and domperidone.

Montelukast Sodium			
Singulair [50]	FDT [51]	ODT [52]	ODT [Present work]
- Mannitol - Microcrystalline cellulose - Hydroxypropyl cellulose - Croscarmellose sodium - Aspartame - Red ferric oxide - Cherry flavor - Magnesium stearate	- Mannitol - Microcrystalline cellulose - Crospovidone ^*^ - Sodium starch glycolate ^*^ - Sodium saccharin - Mint flavor - Talc - Magnesium stearate	- Mannitol - Microcrystalline cellulose - Crospovidone or - Croscarmellose sodium - Aspartame - Talc - Magnesium stearate	- Mannitol ^+^ - Chitin ^+^ - Strawberry flavor - Aspartame - Sodium stearyl fumarate
**Domperidone**			
**Oroperidys [53]**	**FDT [54]**	**FDT [55]**	**FDT [56]**
- Mannitol - Microcrystalline cellulose - Maltodextrin - Croscarmellose sodium - Glucose - Mint flavor - Peppermint gasoline - Anise oil - Gasoline mint - Levomentol - Glycyrrhizate ammonium - Gasoline cloves - Acesulfame potassium - Arabic gum - Sulphur dioxide - Magnesium stearate	- Mannitol - Microcrystalline cellulose - Sodium starch glycolate - Lactose - Talc - Magnesium stearate	- Mannitol - Microcrystalline cellulose - Sodium starch glycolate - Lactose - Crospovidone - Aspartame - Citric acid - Sodium bicarbonate - Maize starch - Starch 1500 - Colloidal anhydrous silica - Lemon flavor - Green lake color - Menthol - Magnesium stearate	- Mannitol - Microcrystalline cellulose - Camphor - Ispaghula husk - Povidone K30 - Crospovidone - Guar gum - Aspartame - Colloidal anhydrous silica - Orange flavor - Talc - Magnesium stearate

* Co-processed together by alcoholic granulation, ^+^ Co-processed together by roll compaction, FDT: fast disintegrating tablet, ODT: oro-dispersible tablet.

**Table 5:** Composition and physical properties of montelukast and domperidone oro-dispersible tablets.

**Material**	**Composition (% w/w)**					
	**Montelukast**			**Domperidone**		
Drug	01.77			03.40		
**Cop–CM**	94.73			93.10		
Strawberry powder flavor	02.00			02.00		
Aspartame	00.50			00.50		
Sodium stearyl fumarate	01.00			01.00		
**Tablet Physical Properties**	**Tablet preparation process**					
	**DC**	**RC**	**WG**	**DC**	**RC**	**WG**
Crushing force (N)	70–80			60–70		
Friability (%)	0.25	0.32	0.30	0.18	0.29	0.37
Disintegration time(s)	20	30	20	26	20	28

DC, RC and WG represent direct compression, roll compaction and wet granulation, respectively. **Cop–CM** represents the co-processed chitin-mannitol (2:8 w/w) mixture.

### 2.10. Stability Studies

The stability of montelukast and domperidone tablets prepared by RC procedures in Section 2.9 (functionality) was investigated. Tablets were packed in aluminum/aluminum packs and incubated at 25 °C and 40 °C/75% relative humidity for different periods of time and then tested by HPLC methods. The HPLC method was initially tested for system suitability (*i.e.*, peak symmetry, repeatability, and resolution) and for validation parameters (*i.e.*, specificity, stability in solution, linearity and limit of quantitation (LOQ)) according to USP [57] and ICH guidelines [58]. The HPLC results showed a good separation between the drugs and their related impurities. Also, the methods were found to be suitable for stability studies, *i.e.*, the resolution, tailing factors and injection repeatability are within the acceptable criteria. In addition, analysis of samples obtained from stress testing studies in solutions (0.1 N NaOH, 0.1 N HCl, and 0.3% H_2_O_2_) indicated that the HPLC methods are stability indicating. The methods were linear over the range of ±50% of the target concentrations with *r*^2^ of > 0.99. The LOQ values are within 0.08–0.4 and 0.05–0.14 (w/w %) for montelukast and domperidone tablets, respectively.

**Table 6:** Stability data for montelukast and domperidone oro-dispersible tablets.

Product	Compound/Limit	Percentage (w/w)			
		25 °C/65%RH		40 °C/75% RH	
		Initial	24 Months	3 Months	6 Months
Montelukast	Drug/90%–110%	99.4	104.8	102.8	98.2
	Drug S–oxide/≤2.0%	0.9	1.1	3.4	3.6
	Drug *cis*–isomer/≤0.5%	0.1	0.5	0.1	0.1
	Any other ≤ 0.4%	0.0	0.2	0.1	0.1
	Total impurities ≤ 3.0%	1.0	2.0	3.6	3.8
Domperidone	Drug/95%–105%	100.1	–	100.7	101.4
	Any other/≤0.25%	0.06	–	0.04	0.04
	Total impurities/≤0.5%	0.2	–	0.1	0.1

As shown in the data in Table 6, no significant decrease in the potency of montelukast and domperidone tablets occurred upon storage at 40 °C/75% RH for six months. Montelukast is liable to oxidation by heat [59] forming montelukast S-oxide; however, under shelf–life conditions (25 °C/65% RH) the degradation content of this product is within the limit (2.0%). Domperidone tablets display excellent stability when the fraction of impurities does not exceed 0.1% after 6 months incubation at 40 °C/75% RH. Both products showed fast disintegration (<30 s) and drug release (>90%) before and after incubation at 40 °C/75% RH indicating the absence of formulation ageing (Table 6).

## 3. Experimental Section

### 3.1. Materials

Α-Chitin of average molecular weight 1000 KD, degree of acetylation of about 0.96 and a mean particle size of 90 μm (Zhejiang Jiande Biochemical, Jiande, China) and d-mannitol (Pearlitol), crystalline grade, with a mean particle size of 160 μm (Roquette, Lestrem, France) were used, Purified water of BP grade, Mannogem EZ and Pharmaburst C1 (SPI, Septemes-Les Vallons, France), Isomalt galenIQ™ 721 (BENEO–Palatinit GmbH, Mannheim, Germany), PanExcea MHC200G (Avantor Performance Materials, Inc./Center Valley, PA, USA), sodium stearyl fumarate (JRS, Patterson, NY, USA) and Crospovidone (Polyplasdone XL) with an average particle size of 110–140 (ISP, Wayne, NJ, USA) were also used. All active pharmaceutical ingredients employed *i.e.*, montelukast sodium (Mylan Lab., Hyderabad, India), domperidone (Xinshiji pharma, Fuzhou, Fujian, China) and metronidazole (Hubei Max Pharma, Wuhan, Hubei, China) were of pharmaceutical grade. All other excipients and reagents used were of pharmaceutical or analytical grades, respectively.

### 3.2. Methods

#### 3.2.1. Preparation of co-Processed Mannitol-Chitin Excipient

Three co–processed mixtures (each 1 kg) of chitin and mannitol of different ratios (1:9, 2:8 and 3:7 w/w) were prepared using different processing techniques, *i.e.*, direct mixing, RC and WG.

*Direct mixing:* The three mixtures were separately passed through a 1000 μm mesh sieve (Fritsch, Idar-Oberstein, Germany) and then mixed for 5 min at 10 rpm using a 1 L cubic blender equipped with a motor drive machine (Erweka, Heusenstamm, Germany).

*Roll compaction*: The three mixtures prepared by direct mixing were compacted using a roll compactor equipped with DPS type rolls (TFC–labo, Vector Corporation, Marion, IA, USA), set at about 5 MPa roll pressure, 4 rounds/minutes roll speed and 20 rounds/minutes screw control speed. The compacted powders were collected and passed through either the 710 μm or 1000 μm sieves using a milling machine equipped with a motor drive machine. Finally the granules were mixed for 5 min at 10 rpm using a 1-L cubic blender equipped with a motor drive machine.

*Wet granulation:* 350, 450 and 550 mL of 14.5% (w/v) of aqueous mannitol solutions were used as granulating agents to prepare the chitin-mannitol mixtures (1:9, 2:8 and 3:7 w/w ratio, respectively). The sieved chitin and the remaining quantity of mannitol were placed in granulation pans (Erweka, Heusenstamm, Germany) and granulated with the mannitol solution using a mixing speed of 150 rpm. The wet masses were passed through a 9.5 mm sieve. Drying was performed at 60 °C using a drying oven (UT6200, Heraeous, Hanau, Germany). The granules were passed through the 1000 μm sieve and mixed using the same procedures as used for the RC preparation.

#### 3.2.2. Characterization of Co-Processed Chitin-Mannitol (**Cop–CM**)

*Fourier transform infrared spectroscopy:* FT-IR measurements were undertaken using an FT-IR instrument (Paragon 1000, Perkin Elmer, Llantrisant, UK) by means of thin pellets containing 1 mg of each sample dispersed in 100 mg of KBr. The spectra were recorded at room temperature as an average of 30 scans, in the 400–4000 cm^−1^ range with a spectral resolution of 1 cm^−1^. In order to minimize the effects of traces of CO_2_ and water vapour from the atmosphere of the sample compartment, the spectrometer was purged with nitrogen.

*X-ray powder diffractometry:* The XRPD patterns were measured using an X-ray diffractometer (PW1729, Philips, Amsterdam, The Netherlands). The radiation was generated using a *CoK*α source and filtered through Ni filters; a wavelength of 1.79025 Å at 40 mA and 35 kV was used. The instrument was operated over the 2θ range of 5–60°. The range and the chart speed were set at 2 × 103 cycles/s and 10 mm/2θ, respectively.

*Differential scanning calorimetry (DSC)*: Samples (~5 mg) were hermetically sealed in aluminum pans and scanned over a range temperature of 0–300°C at a rate of 5 °C/min (DSC 25, Mettler Toledo, Greifensee, Germany). The instrument was calibrated using indium and the calorimetric data were analyzed using STAR software (version 9).

*Scanning electron microscopy*: The morphology of the samples were determined using a scanning electron microscope (Quanta 200 3D, FEI, Eindhoven, Netherland) operated at an accelerating voltage of 1200 V. The sample (0.5 mg) was mounted onto a 5 × 5 mm silicon wafer affixed via graphite tape to an aluminum stub. The powder was then sputter-coated for 105 s at a beam current of 20 mA/dm^3^ with a 100 Å layer of gold/palladium alloy.

*Angle of Repose:* Angle of Repose (*q*) was determined using the funnel method [37]. The sample blends were poured through a funnel that could be raised vertically until a maximum cone height (*h*) was obtained. Radius of the heap (*r*) was measured and then *q* was calculated using the formula:

(5) $$q=\tan^{-1}(\frac{h}{r})$$

*Particle size:* The particle size distributions for all samples were measured by using a Malvern Mastersizer 2000 instrument (Malvern Instruments Ltd., Worcestershire, UK). Approximately 5 mL of powder was used for each measurement. The air pressure was set at 2.0 bar, and the feed rate was set at 50%. The particle size distributions D10, D50 and D90 were recorded. Each sample was measured three times.

*Bulk density (BD) and tapped density (TD):* Approximately 100 mL of powder was gently poured into a tarred graduated cylinder and the initial volume and weight of the material recorded. The graduated cylinder is placed on a tap density tester (SVM, Tapped volumeter, Erweka, Heusenstamm, Germany) and the final volume is recorded after 200 taps. *BD* and *TD* are calculated by dividing the initial and final volume of powder by the weight of powder, respectively [37].

*Water content:* The water content of the powders was measured using a Karl Fischer Titrator (DL38, Mettler Toledo, Greifensee, Switzerland).

*pH measurement:* The pH was measured using a a pH-meter (3030, Jenway, Staffordshire, UK).

*Hygroscopicity:* Samples (2.5 g) were stored in desiccators containing water saturated salt solutions at room temperature (20 °C) for 10 and 14 days. The media compositions were set according to the Handbook of Chemistry and Physics [60] to obtain relative humidity’s (RHs) of 52%, 62%, 75%, 84% and 95% using Ca(NO_3_).4H_2_O, NH_4_NO_3_, NaCl, KCl and Na_2_HPO_4_.12H_2_O, respectively. The samples were withdrawn after a fixed time period and kept at 20 °C for 1 and 24 h before weighing and calculating the fractional gain in mass compared to the original mass under the different RH conditions.

#### 3.2.3. Physical and Chemical Properties of Tablets Prepared from **Cop–CM**

*Crushing force, disintegration and friability*: The crushing force (6D, Schelenuiger, Thun, Switzerland), disintegration (2T31, Erweka, Heusenstamm, Germany) and friability (Erweka, Heusenstamm, Germany) tests were performed following the general tests in the BP [42].

*Influence of particle size on tablet properties:* Samples of **Cop–CM** powders were passed through either 710 μm or 1000 μm sieves and individually mixed with 1.0% (w/w) of sodium stearyl fumarate as a lubricant and then compressed using a single punch tabletting machine (SF3, Chadmach Machinery, Ahmedabad, India) at different crushing force values of 30, 50, 70, 90, 110, 130 and 150 N, using a 10 mm circular punch to produce tablets of 250 mg weight. The disintegration time and friability were measured at each tablet crushing force point. Some of the commercially available ODT bases (Pharmaburst C1, Isomalt galenIQ™ 721, Mannogem EZ and PanExcea MHC 200G) in addition to Mannogem EZ and 3% crospovidone, Isomalt galenIQ™ 721 and 3% crospovidone powders were lubricated using 1% w/w sodium stearyl fumarate and used as reference materials.

*Moisture uptake studies:* Moisture uptake studies were conducted in order to assess the physical stability of the ODTs composed of **Cop–CM** base. Twenty tablets were kept in a desiccator over calcium chloride at 37 °C for 24 h. The tablets were then weighed and exposed to 75% relative humidity, at room temperature for 14 days. The required humidity was achieved by the use of saturated sodium chloride solution at the bottom of the desiccators for 72 h. Tablets were weighed and the fractional increase in mass was recorded [60].

*Wetting time:* A piece of tissue paper (8 cm in diameter), folded twice was placed in a Petri dish (8.5 cm in diameter) containing 6 mL of water. One tablet was carefully placed on the surface of the tissue paper and allowed to wet completely [60]. The time required for the water to reach the upper surface of the tablet was recorded as the wetting time.

*Loading capacity:* A study was undertaken to measure the impact of the drug load on the performance of **Cop–CM** as tablet excipient. Tablets were prepared by DC using a SF3 single punch tablet press machine equipped with D-type tooling. The seven experiments were performed using **Cop–CM** and metronidazole, containing, 0%, 10%, 30%, 50%, 70%, 90% and 100% (for comparison) of metronidazole lubricated with 0.3% sodium stearyl fumarate. The prepared tablets were circular in shape with a diameter of 12 mm and a mass of 500 mg. Tablets from each experiment were evaluated for crushing force and disintegration time.

*Functionality*: To investigate the functionality of **Cop–CM**, two drug models as small strength tablets were studied including montelukast sodium and domperidone. The tablets for the two drugs were prepared by DC, RC as well as WG methods. The montelukast and domperidone tablets contained 1.77% and 3.4% of drug and 94.7% and 93.1% of **Cop–CM**, respectively. In addition, both drugs formulations contained strawberry powder (2.0%), aspartame (0.5%) and sodium stearyl fumarate (1.0%). For the DC experiments, montelukast and all excipients, except sodium stearyl fumarate, were first mixed for 2 min and then sodium stearyl fumarate was added and further mixed for another 2 min.

For the RC procedure, montelukast (1.77% w/w), **Cop–CM** (25% w/w, intra–granular) and sodium stearyl fumarate (0.6% w/w) were compacted by employing a DP roll type compaction using 10 MPa roll pressure, 3 rounds/minutes roll speed and 43 rounds/minutes screw control speed and then passed through a 1000 μm sieve. The remaining amount of **Cop–CM** was added and mixed for 2 min followed by the addition of sodium stearyl fumarate (0.4% w/w). The powder was then further mixed for 2 min.

For the WG procedure, montelukast (1.77% w/w) and **Cop–CM** (35% w/w, intra-granular) were granulated with 50% (w/v) ethanol in water, dried at 60 °C, and then sieved using the 1000 μm sieve. The remaining amount of **Cop–CM** was added and mixed for 2 min; sodium stearyl fumarate (1%) was then added and mixed for a further 2 min. The same procedures were repeated for domperidone. The prepared mixtures for each drug were compressed at 300 mg tablet weight, for which 10 mm shallow concave punches and dies were used. Dissolution tests (DT–80; Erweka, Heusenstamm, Germany) for montelukast and domperidone were performed according to the USP [61], FDA [62] and BP [63] published dissolution methods. The released fraction of montelukast and domperidone were determined spectrophotometrically (Du-650i, Beckman Coulter, Brea, CA, USA) by measuring the first derivative absorbance modes at 290 nm for montelukast and absorbance mode at 280 nm for domperidone.

*Compressibility:* Mannitol, chitin and **Cop–CM** powder samples were compressed using a universal testing machine (RKM 50, PR–F system, ABS Instruments, Tamilnadu, Germany) equipped with 12 mm round, flat face upper and lower punches as well as dies; punch speed was fixed at 10 mm/min. Different compression forces from 80 to 390 MPa were applied. Three tablets were prepared to ensure reproducibility. Compression was carried out at 400 mg tablet weight. The compression behavior of the samples was evaluated using Kawakita analysis [43,44,45,46].

*Stability studies:* Montelukast and domperidone tablets prepared by DC were packed in aluminum/aluminum strips and stored at 25 °C/60% RH and 40 °C/75% RH for 24 and 6 months, respectively. At different interval times, tablets were withdrawn and tested for dissolution and content of drug and its related substances by stability indicating and validated HPLC methods [57,59,64]. The HPLC instrument was equipped with a P1000 pump and a UV1000 detector (TSP, Alexandria, VA, USA). For montelukast tablets, a mixture of acetate buffer (0.385% ammonium acetate in water adjusted with acetic acid to pH 3.5) and methanol (15:85, v/v) was used as the mobile phase and an octadecylsilyl silica column as the stationary phase (250 × 4 mm, 10 μm). UV detection at 254 nm, a flow rate of 1 mL/min, and 20 μL injection volumes of the test solutions (0.2 mg montelukast /mL of 70% ethanol) were used. While for domperidone tablets, methanol and 0.5% ammonium acetate in water were used as the mobile phases A and B, respectively. A linear gradient elution with a flow rate of 1.5 mL/min was programmed as follows: time 0 min: 30, 70, time 10 min: 100, 0, and time 12 min: 100, 0 for mobile phase A and B, respectively. A based-deactivated, end capped L7 column was used as the stationary phase (Hypersil C8 BDS, 100 × 4.6 mm, 3 μm). UV detection at 280 nm and a 10 μL injection volume of the test solutions (5 mg domperidone/mL of 0.1 M·HCl in 50% ethanol) were employed. The HPLC method was initially tested for system suitability (*i.e.*, peak symmetry, repeatability, and resolution) and for validation parameters (*i.e.*, specificity, recovery, stability in solution, linearity, and limit of quantitation (LOQ)) according to USP guidelines [57].

## 4. Conclusions

Co-processing of crystalline mannitol with α-chitin by RC offers an excellent multifunctional base for ODT formulations. The novel excipient displayed fast disintegration and wetting properties over a wide range of tablet crushing force values in comparison with commercially available ODT bases. Regardless of the preparation method (DC, WG or DG), the functionality of the novel excipient was preserved. Moreover, the excipient can accommodate a high amount of drug without affecting its functionality. Utilization of the novel excipient in ODT containing active pharmaceutical ingredients offers very fast disintegration and wetting rates, excellent chemical stability and binding properties. Consequently, the need to use expensive packaging materials for ODT will be eliminated as tablets will no long be prone to breaking during patient use.
